# Digitally Delivered Exercise and Education Treatment Program for Low Back Pain: Longitudinal Observational Cohort Study

**DOI:** 10.2196/38084

**Published:** 2022-06-21

**Authors:** Helena Hörder, Håkan Nero, Majda Misini Ignjatovic, Ali Kiadaliri, L Stefan Lohmander, Leif E Dahlberg, Allan Abbott

**Affiliations:** 1 Department of Clinical Sciences Lund Orthopaedics Lund University Lund Sweden; 2 Department of Science Joint Academy Malmö Sweden; 3 Clinical Epidemiology Unit, Department of Clinical Sciences Lund Orthopaedics Lund University Lund Sweden; 4 Unit of Physiotherapy, Department of Health Medicine and Caring Science Linköping University Linköping Sweden

**Keywords:** low back pain, telehealth, physiotherapy, digital care, exercise, rehabilitation, back pain, pain management, telemedicine, digital therapy, chronic pain, health outcome

## Abstract

**Background:**

Exercise and education is recommended as first-line treatment by evidence-based, international guidelines for low back pain (LBP). Despite consensus regarding the treatment, there is a gap between guidelines and what is offered to patients. Digital LBP treatments are an emerging way of delivering first-line treatment.

**Objective:**

The aim of this study is to evaluate outcomes after participation in a 3-month digitally delivered treatment program for individuals with subacute or chronic LBP.

**Methods:**

We analyzed data from 2593 consecutively recruited participants in a digitally delivered treatment program, available via the national health care system in Sweden. The program consists of video-instructed and progressive adaptable exercises, education through text lessons, and a chat and video function connecting participants with a personal physiotherapist. The primary outcome was mean change and proportion reaching a minimal clinically important change (MCIC) for LBP (2 points or 30% decrease) assessed with the numerical rating scale (average pain during the past week, discrete boxes, 0-10, best to worst). Secondary outcomes were mean change and proportion reaching MCIC (10 points or 30%) in disability, assessed with the Oswestry Disability Index (ODI; 0-100, best to worst) and a question on patient acceptable symptom state (PASS).

**Results:**

The mean participant age was 63 years, 73.85% (1915/2593) were female, 54.72% (1419/2593) had higher education, 50.56% (1311/2593) were retired, and the mean BMI was 26.5 kg/m2. Participants completed on average 84% of the prescribed exercises and lessons, with an adherence of ≥80% in 69.26% (1796/2593) and ≥90% in 50.13% (1300/2593) of the participants. Mean reduction in pain from baseline to 3 months was 1.7 (95% CI –1.8 to –1.6), corresponding to a 35% relative change. MCIC was reached by 58.50% (1517/2593) of participants. ODI decreased 4 points (95% CI –4.5 to –3.7), and 36.48% (946/2593) reached an MCIC. A change from no to yes in PASS was seen in 30.35% (787/2593) of participants. Multivariable analysis showed positive associations between reaching an MCIC in pain and high baseline pain (odds ratio [OR] 1.9, 95% CI 1.6-2.1), adherence (OR 1.5, 95% CI 1.3-1.8), and motivation (OR 1.2, 95% CI 1.0-1.5), while we found negative associations for wish for surgery (OR 0.6, 95% CI 0.5-0.9) and pain in other joints (OR 0.9, 95% CI 0.7-0.9). We found no associations between sociodemographic characteristics and pain reduction.

**Conclusions:**

Participants in this digitally delivered treatment for LBP had reduced pain at 3-month follow-up, and 58.50% (1517/2593) reported an MCIC in pain. Our findings suggest that digital treatment programs can reduce pain at clinically important levels for people with high adherence to treatment but that those with such severe LBP problems that they wish to undergo surgery may benefit from additional support.

**Trial Registration:**

ClinicalTrials.gov NCT05226156; https://clinicaltrials.gov/ct2/show/NCT05226156

## Introduction

Low back pain (LBP) is the leading cause of years lived with disability worldwide [1]. Exercise and education is recommended as first-line treatment in clinical guidelines, but ineffective health care resources are too often used, providing low-value or at worst, harmful care [2].

The BetterBack model of care was developed and tested in primary care clinics in Sweden to facilitate guideline implementation [3]. Its biopsychosocial approach includes a face-to-face structured assessment by a physiotherapist (PT), education, and individualized exercises focusing on the core and back muscles. In a stepped-clustered randomized study, participants in the program did not differ in pain and disability compared to a group receiving routine physiotherapy care but reported higher satisfaction along with clinically meaningful improvement in LBP illness perception and quality of life [4].

Telehealth, defined as the “delivery of healthcare at a distance using information and communication technology” (ICT) has been rapidly adopted during the COVID-19 pandemic [5,6]. It may help overcome barriers in traditional face-to-face interventions, such as limited access, low adherence, lack of flexibility, and travel costs [7-9]. Systematic reviews suggest that ICT increases exercise adherence and may provide pain and function improvements similar to or better than those provided by face-to-face treatment for a variety of musculoskeletal (MSK) conditions [10-13].

In digital LBP treatment, published results showed considerable heterogeneity between studies with possible positive effects on pain and disability in the short-term [13-18]. However, sample sizes were small with participants being predominantly of working age.

To our knowledge, this study is the first to report real-world data collected from an LBP treatment app that is part of a public health care system. The aim is to evaluate change and proportion of responders in pain as a primary outcome, and disability and patient acceptable symptom state (PASS) as secondary outcomes; and to examine if sociodemographic, baseline health, and treatment-related factors are associated with pain reduction.

## Methods

### Ethics Approval

This was a longitudinal observational cohort study with consecutively recruited participants, approved by the Swedish Ethical Review Authority (diary #2021-04183, 2021-12-20) and registered at ClinicalTrials.gov (NCT05226156). Digital informed consent was obtained from participants at registration. The study adheres to the STROBE (Strengthening the Reporting of Observational Studies in Epidemiology) guidelines for observational studies [19].

### Sample

**Figure 1:**
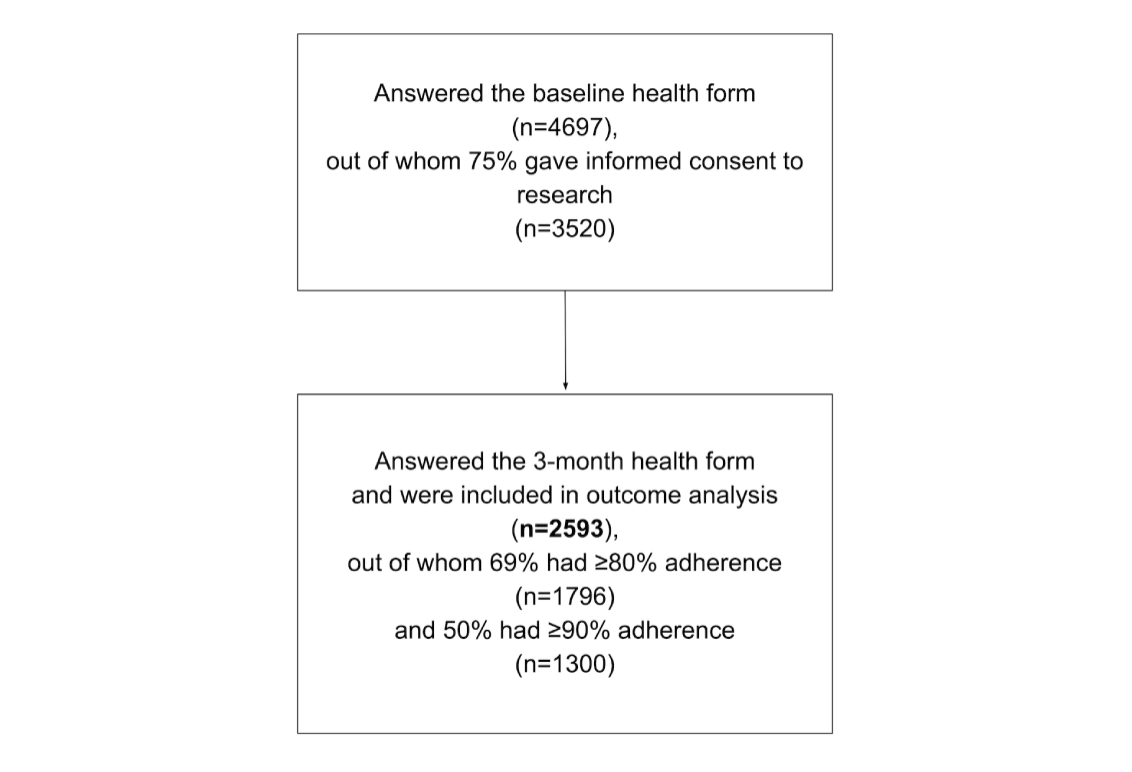
Flowchart of participants in the digital treatment for low back pain.

Data were extracted from the database on March 16, 2022, and included all people that had given their informed consent and initiated their LBP treatment between April 27, 2021, and December 16, 2021 (Figure 1).

Participants joined the Joint Academy (JA) program on their own initiative via online advertisements and campaigns placed on search engines and social networks through recommendation by their local PT or general practitioner, or through their insurance company. Inclusion criteria for treatment were the following: an age >18 years and presence of subacute or chronic LBP (including nonspecific LBP, disc degeneration, spondylosis, spinal stenosis [20], olisthesis). Participants without a prior clinical diagnosis of nonspecific LBP (diagnosis code ICD-10 M54.5) required a clinical diagnosis confirmed by a PT via telephone or video call. In the app, participants first need to negate recent trauma within 0 to 6 months and symptoms of cauda equina syndrome in order to be registered in the program. At the start-up consultation with the PT, further exclusion criteria were considered before eligibility: malignant disease with or without suspected metastasis, fracture or vertebral compression within 6 months, and infection. If there were uncertainties regarding diagnosis or comorbidities, candidate participants were recommended to seek face-to-face care before inclusion in the program. Additional relative exclusion criteria were assessed by the PT: previous or current cancer or involuntary weight loss, radiculopathy below the knee, opioid-demanding pain or pain while resting, inflammatory back pain, pregnancy or postpregnancy, and older participants (>75 years) with multiple diseases and/or structural deformities (eg, scoliosis).

### The Digital Treatment Program

The treatment program is available via the national health care system for all residents in Sweden. The procedure is similar to that of other JA (see Multimedia Appendix 1) programs managing osteoarthritis and MSK ailments [21,22]. The digital LBP program was inspired by the face-to-face BetterBack model of care [3,4].

Briefly, the program consists of a mobile app with 2 daily distributed individualized and progressively adaptable video exercises, focusing on strengthening the lower back, glutes, and core musculature. Short sessions of patient education are also delivered 2-3 times per week, followed by a quiz question to ensure the information has been understood properly. Correctly answering the quiz is mandatory to be able to continue the program. The program offers a peer-support chat room, and a registered PT supervises the participant and is available through a continuous asynchronous chat function during the full participation period. The program also contains 3 compulsory telephone or video consultations with the PT, 1 at the start, 1 after 6 weeks, and 1 after 3 months.

### Variables

All participants answered relevant sociodemographic questions at baseline including those regarding sex, education, and work situation, using the question *“*Which alternative describes your current situation best?*”* (working, studying, sick leave full-time, sick leave part-time, retired, unemployed); weight and height, pain in other joints, and general health, using the question “Mark on the scale how good or bad your current health is?” as assessed with the numerical rating scale (NRS; 0-10,worst imaginable to best imaginable); anxiety or depression according to the EQ-5D-5L (level 1-5, no problems to severe problems) [23]; medications, using the question “In the past months, have you taken any medication for the pain in your lower back ?”(yes or no); wish to undergo surgery, using the question “Are your symptoms so severe that you wish to undergo surgery?” (yes or no); physical activity, using the question “How much time do you spend in a typical week on daily physical activity that is not exercise, such as walking, cycling or gardening?” (7-grade scale: 0, <30, 30-60, 60-90, 90-150, 150-300, >300 minutes/week) [24]; and motivation or readiness for exercising, using the question “How ready are you to start doing back exercises on a daily basis? (NRS 0-10, not at all ready to extremely ready).

All questions were answered by self-report and collected digitally through the app. Pain was assessed weekly, and a larger health questionnaire was used at baseline and at 3-month follow-up.

### Primary Outcome

LBP was assessed using the NRS (discrete boxes), with the instruction “Mark on the scale your average pain from your lower back in the past week,*”* followed by a 0 to 10–digital scale where 0 indicates “No pain” and 10 indicates “Unbearable” [25]. An absolute improvement in back pain of ≥2 points or a relative improvement of 30% from baseline to 3 months was used to describe a minimal clinically important change (MCIC), in line with practical guidelines toward consensus in reporting MCIC in LBP [26].

### Secondary Outcomes

The Oswestry Disability Index (ODI) version 2.1a was used to assess LBP-related disability. The ODI is divided into 10 sections to assess the level of pain and interference with several activities including sleep, self-care, sex life, social life, and traveling. Each question has 6 possible responses and is scored from 0 to 5 (good to bad). The score for each section is added and divided by the total possible score (50 if all sections are completed), and the resulting score is multiplied by 100 to yield a percentage score with 0% equivalent to no disability and 100% equivalent to a great deal of disability [27]. An absolute improvement of ≥10 points or a relative improvement of 30% from baseline to 3 months was used to describe MCIC, in line with guidelines toward consensus in reporting MCIC in LBP [26].

Radiating pain was assessed using the NRS (discrete boxes), with the instruction “Mark on the scale how much pain you have radiating down your leg,*”* followed by a 0 to 10–digital scale, where 0 indicates “No pain” and 10 indicates “Unbearable” [25].

PASS was assessed at baseline and follow-up with the question: *“*Considering your lower back function, do you feel that your current state is satisfactory? With lower back function you should take into account all activities you have during your daily life, sport and recreational activities, your level of pain and other symptoms, and also your quality of life related to your lower back” (yes or no). The PASS is a treatment-response criterion developed to determine the clinical relevance of a treatment effect [28]. Answering no is referred to as PASS(–), yes is referred to as PASS (+), and changing from no at baseline to yes at 3 months as PASS(–to+).

### Treatment Failure and Adverse Events

If the answer to the PASS question was no, a question of treatment failure was asked at follow-up: “Would you consider your current state as being so unsatisfactory that you think the treatment has failed?” (yes or no).

Adverse events were assessed with the question: “Have you experienced any unwanted side effects of your Joint Academy treatment?” (yes or no). If the answer was yes, a follow-up question was asked: “What type of unwanted side effect?” (choices: severe pain not relieved after 24 hours, a fall or injury during exercising, other).

### Adherence

We defined adherence as the percentage of completed activities out of those delivered to the participants over the course of the treatment period (2 exercises per day and 3-4 educational texts per week). As participants had to check an obligatory box after every exercise and educational text to be able to continue in the program, an estimate of the weekly adherence was available in the dashboard of the treating PT. The commonly used adherence cutoff of ≥80% (in this program referring to performing activities ≥5 days a week) was considered as a lower limit for satisfactory adherence [29].

Information on the number of chat interactions with the PT, initiated either by the PT or the participant, and on if participants chose to take part in an optional peer support group (yes or no) during the treatment was also available through the app.

Dropout was defined as having baseline data and starting the treatment, but not continuing until the 3-month follow-up. The week for the latest registered exercise or educational text was used to define the dropout week.

### Statistical Analysis

To describe the sample, we use mean and SD, frequency, and percentage.

For outcomes at 3 months, we calculated median (percentile), mean (95% CI), and proportions for the total sample and for per protocol samples with ≥80% and ≥90% adherence. The paired *t* test was used to calculate mean change from baseline to 3 months, and McNemar test was used to calculate change in proportions. One-way analysis of variance (ANOVA) was performed in order to detect potential differences in pain reduction at 3 months between groups with different adherence levels (<40%,40%-49%, 50%-59%, 60%-69%, 70%-79%, 80%-89%, and 90%-100%). We also present weekly mean (95% CI) pain during the 3 months, stratified by baseline pain and adherence.

We used univariable logistic regressions to explore variables associated with reaching an MCIC in pain and proportion, reporting a change from no to yes in PASS(–to+). The following variables were selected based on previous research [30,31]: sociodemographic (sex, age, occupational status, educational level), baseline health-related (BMI, NRS LBP, NRS radiating pain, pain medications, wish for surgery, pain in other joints, depression or anxiety, general health, physical activity), and treatment-related (motivation, adherence, interactions with PT, participation in a peer group). For PASS(–to+), we included only those answering no to PASS at baseline (n=2080) and we included reaching MCIC in pain as an independent variable.

We also used multivariable logistic regression, including all variables irrespective of bivariate *P* value. A test for multicollinearity showed variance inflation factor values below 2.5 for all variables, except for age and occupational status. As multicollinearity could be excluded for all other independent variables, they were all included in the multivariate analyses. Odds ratios (ORs) and 95% CIs were calculated and considered statistically significant if the 95% CI did not include 1.

Data analysis was performed using the Python Library Statsmodel version 0.13.2 [32].

## Results

### Participant Characteristics

A total of 4697 individuals answered the baseline questionnaire, of whom 74.94% (3520/4697) had given their informed consent. Out of these, 73.66% (2593/3520) answered the 3-month questionnaire and were included in the outcome analyses (Figure 1). Mean participant age was 63 years, 73.85% (1915/2593) were female, 54.72 % (1419/2593) had a university level education, and 50.56% (1311/2593) were retired (Table 1).

**Table 1:** Characteristics of participants in digitally delivered exercise and education treatment for low back pain (N=2593).

Variables			Values
**Sociodemographic characteristics**			
	Female, n (%)		1915 (73.85)
	Age (years), mean (SD)		63.0 (11.0)
	**Educational level, n (%)**		
		Have not graduated high school	221 (8.52)
		Graduated high school	953 (36.75)
		College/university degree	1419 (54.72)
	**Occupational status, n (%)**		
		Working	1093 (42.15)
		Studying	20 (0.77)
		Sick leave full-time	67 (2.58)
		Sick leave part-time	47 (1.81)
		Retired	1311 (50.56)
		Unemployed	55 (2.1)
**Baseline health-related characteristics**			
	BMI (kg/m^2^), mean (SD)		26.5 (4.4)
	Baseline pain, NRS^a^ (0-10), mean (SD)		4.9 (1.9)
	Reported radiating pain (>0 NRS), n (%)		1630 (62.86)
	Pain medications for back pain during last month, yes, n (%)		1252 (59.36)^b^
	Problem severity such that surgery is desired, n (%)		138 (5.32)
	Presence of pain in other joints, n (%)		1956 (75.43)
	Depression or anxiety (any problem = level 2-5 EQ-5D-5L), n (%)		1351 (52.10)
	General health, NRS (0-10), mean (SD)		6.2 (1.6)
	Physical activity level, ≥150 min/week, n (%)		1065 (40.73)
	Motivation/readiness ruler to start exercising (NRS 0-10, not at all to extremely), mean (SD)		9.3 (1.3)
**Treatment-related characteristics**			
	**Adherence to treatment during 3 months:**		
		Proportion of daily exercises/educational texts completed, mean (SD)	83.9 (17.0)
		≥80% adherence, n (%)	1796 (69.26)
		≥90% adherence, n (%)	1300 (50.13)
	**Number of chat interactions with the PT^c^ during the treatment**		
		Messages received from the PT, mean (SD)	21 (12)
		Messages sent to the PT, mean (SD)	9 (7)
	Participated in peer support group, n (%)		866 (33.40)

^a^NRS: numerical rating scale.

^b^Due to technical issues in the app, the total is 2109.

^c^PT: physiotherapist.

Dropouts (ie, those who did not continue up to the 3-month follow-up) accounted for 26.34% (927/3520) of the total baseline sample (see Multimedia Appendix 2 for graph of dropouts per week). Compared to the total sample, dropouts differed in most baseline- and treatment-related characteristics. For example, they were more often of working age, more often reported problems with depression or anxiety, and had a lower physical activity level at baseline; furthermore, a lower proportion participated in a peer group during the treatment (91/927, 9.82% vs 866/2593, 33.40%; *P*<.001; see Multimedia Appendix 2 for comparison of baseline characteristics).

### Adherence

During the 3-month treatment, participants completed on average 84% of the daily exercises and educational texts. An adherence of ≥80% (corresponding to at least 5 days/week) was seen in 69.26% (1796/2593) and an adherence of ≥90% (corresponding to 6-7 days/week) in 50.13% (1300/2593; Table 1). Those with ≥90% adherence compared to those with <90%, were older, more often retired, had lower BMI, and less often reported problems with anxiety or depression at baseline; meanwhile, we observed no difference relative to sex or educational level (see Multimedia Appendix 2 for comparison of baseline characteristics).

### Outcomes at 3 Months

The median reduction in LBP from baseline to 3 months was an NRS of 2 points, and the mean reduction was NRS 1.7 (95% CI –1.8 to –1.6) points, corresponding to a 35% relative change. The mean reduction for ODI was 4.1 (95% CI –4.5 to –3.7) points, corresponding to a 16% relative change, and the mean reduction in radiating pain was NRS 0.6 (95% CI –0.7 to –0.5). An MCIC in LBP (defined as either NRS ≥ –2 points or 30% relative reduction) was seen in 58.50% (1517/2593) of participants, while for ODI (defined as either ≥–10 points or 30% relative reduction), an MCIC occurred in 36.48% (946/2593). A total of 46.24% (1199/2593) reported yes to PASS(+) at 3 months, and 30.35% (787/2593) reported a change from no to yes in PASS(–to+; Table 2).

**Table 2:** Change in outcomes from baseline to 3-month follow-up among participants in digitally delivered exercise and education treatment for LBP.^a^ Results are for total sample (N=2593) and for subgroups with ≥80% (n=1796) and ≥90% adherence (n=1300).

			Baseline	3-month follow-up	Change
**LBP, NRS^b^**					
	**Mean (95% CI)**				
		Total sample	4.9 (4.8 to 5.0)	3.2 (3.1 to 3.3)	–1.7 (–1.8 to –1.6)
		≥80% adherence	4.9 (4.8 to 5.0)	3.0 (2.9 to 3.1)	–1.8 (–1.9 to –1.8)
		≥90% adherence	4.9 (4.8 to 5.0)	3.0 (2.9 to 3.1)	–1.9 (–2.0 to –1.8)
	**Median (Q^c^ 1-Q3)**				
		Total sample	5.0 (3.0 to 6.0)	3.0 (2.0 to 4.0)	–2.00
		≥80% adherence	5.0 (3.0 to 6.0)	3.0 (2.0 to 4.0)	–2.00
		≥90% adherence	5.0 (3.0 to 6.0)	3.0 (2.0 to 4.0)	–2.00
**ODI^d^**					
	**Mean (95% CI)**				
		Total sample	25.5 (25.0 to 26.0)	21.4 (20.9 to 21.9)	–4.1 (–4.5 to –3.7)
		≥80% adherence	25.3 (24.7 to 25.9)	21.0 (20.4 to 21.6)	–4.3 (–4.8 to –3.9)
		≥90% adherence	25.3 (24.6 to 26.0)	20.9 (20.2 to 21.6	–4.4 (–4.9 to –3.8)
	**Median (95% CI)**				
		Total sample	24.0 (16.0 to 34.0)	20.0 (12.0 to 30.0)	–4.00
		≥80% adherence	24.0 (16.0 to 34.0)	20.0 (12.0 to 30.0)	–4.00
		≥90% adherence	24.0 (16.0 to 34.0)	20.0 (12.0 to 30.0)	–4.00
**Radiating pain, NRS**					
	**Mean (95% CI)**				
		Total sample	2.3 (2.2 to 2.4)	1.7 (1.6 to 1.8)	–0.6 (–0.7 to –0.5)
		≥80% adherence	2.3 (2.2 to 2.4)	1.6 (1.5 to 1.7)	–0.7 (–0.6 to –0.8)
		≥90% adherence	2.3 (2.2 to 2.4)	1.6 (1.5 to 1.7)	–0.7 (–0.6 to –0.8)
	**Median (95% CI)**				
		Total sample	2.0 (0.0 to 4.0)	1.0 (0.0 to 3.0)	–1.00
		≥80% adherence	2.0 (0.0 to 4.0)	1.0 (0.0 to 3.0)	–1.00
		≥90% adherence	2.0 (0.0 to 4.0)	1.0 (0.0 to 3.0)	–1.00
**Reaching an MCIC^e^ in LBP, n (%)**					
	Total sample		N/A^f^	1517 (58.50)	N/A
	≥80% adherence		N/A	1124 (62.58)	N/A
	≥90% adherence		N/A	833 (64.08)	N/A
**Reaching an MCIC in ODI, n (%)**					
	Total sample		N/A	946 (36.48)	N/A
	≥80% adherence		N/A	671 (37.36)	N/A
	≥90% adherence		N/A	484 (37.23)	N/A
**Patient acceptable symptom state, n (%)**					
	Total sample		513 (19.78)	1199 (46.24)	787 (30.35)^g^
	≥80% adherence		363 (20.21)	852 (47.44)	556 (30.96)^g^
	≥90% adherence		279 (21.26)	647 (49.77)	419 (32.23)^g^
**Considered treatment failed, n (%)**					
	Total sample		N/A	117 (4.51)	N/A
	≥80% adherence		N/A	75 (4.18)	N/A
	≥90% adherence		N/A	44 (3.38)	N/A
**Adverse events yes, n (%)**				63 (2.43)	
	**Event type, n (%)**				
		Pain more than 24 h	N/A	16 (25.81)	N/A
		Fall/injury	N/A	1 (1.61)	N/A
		Other	N/A	45 (72.58)	N/A

^a^LBP: low back pain.

^b^NRS: numerical rating scale; score range 0 to 10 (best to worst).

^c^Q: quartile.

^d^ODI: Oswestry Disability Index; 0% to 100% (no disability to a great deal of disability).

^e^MCIC: minimal clinically important change; taken from Ostelo et al (26); pain NRS = absolute improvement of ≥2 points or relative improvement of 30%; ODI = absolute improvement ≥10 points or relative improvement of 30%.

^f^N/A: not applicable.

^g^Change in patient acceptable symptom state refers to the proportion that changed from no at baseline to yes at 3-month follow-up.

### Pain Reduction Relative to Adherence and Pain at Treatment Start

**Figure 2:**
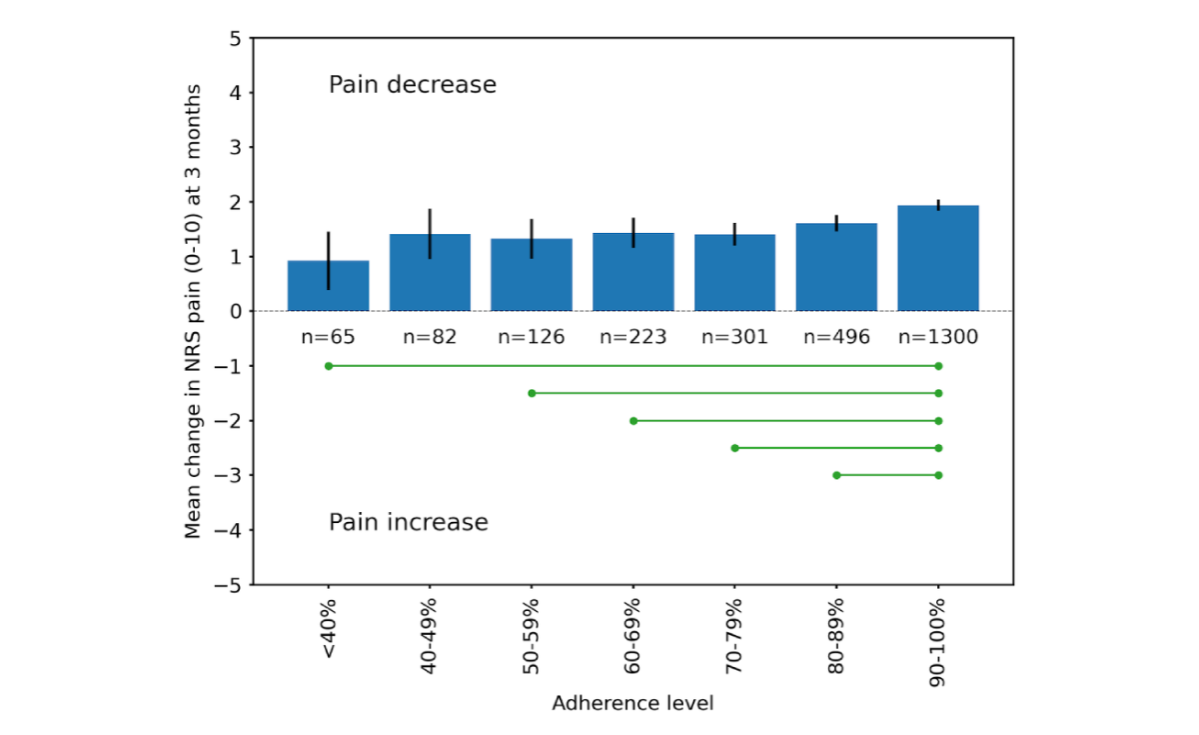
Mean pain reduction from baseline to 3 months stratified by adherence to treatment. Green lines with dots show statistically significant pairs (analysis of variance *P*<.05) with all other pairs being nonsignificant. NRS: numerical rating scale.

Those with ≥90% adherence had a greater mean pain reduction at 3 months compared to those with <90% adherence. The difference compared to those with 80%-90% adherence was small but statistically significant with a mean pain reduction of 1.9 (95% CI –2.0 to –1.7) versus 1.6 (95% CI –1.7 to –1.5; Figure 2). We observed no differences in mean pain reduction between those with 80%-90% and those with <80% adherence. The lowest pain reduction was seen among those with <40% adherence (0.9; 95% CI –1.5 to –0.4), with a similar pain reduction of 0.9 among dropouts at their last weekly measure before dropping out (95% CI –1.1 to –0.7; Figure 2).

**Figure 3:**
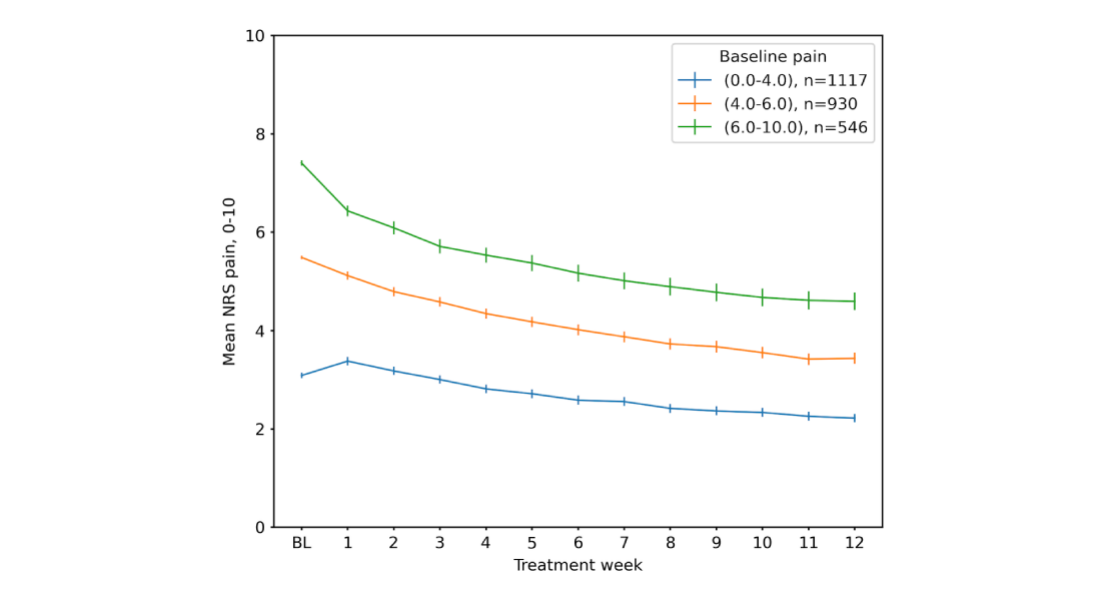
Weekly mean (95% CI bars) pain (NRS 0-10) during 3 months' participation in digitally delivered exercise and education treatment stratified by baseline pain. BL: baseline; NRS: numerical rating scale.

**Figure 4:**
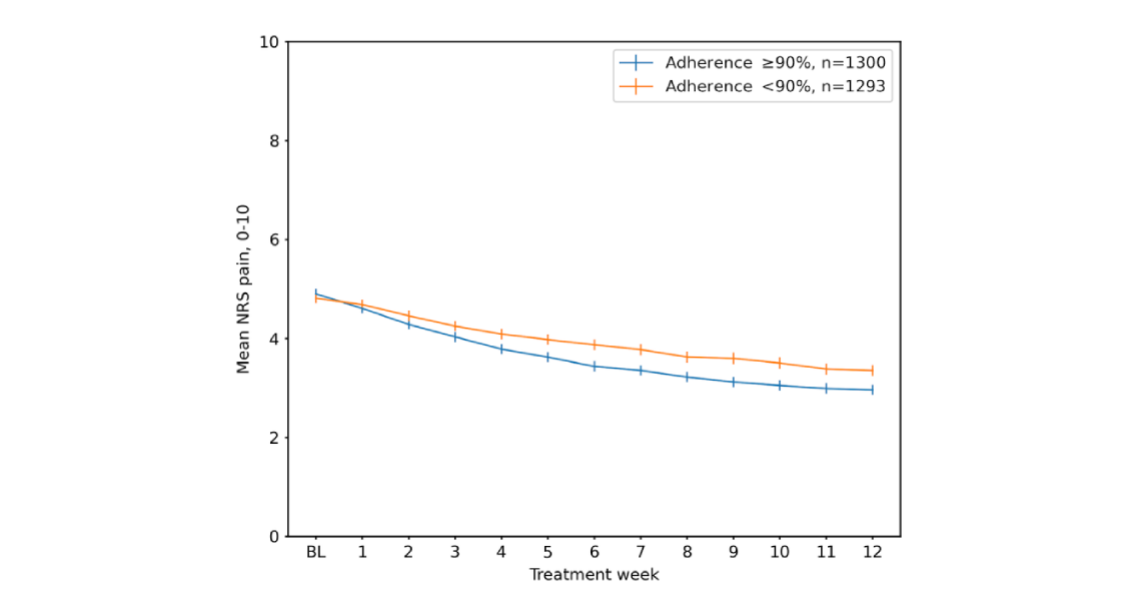
Weekly mean (95% CI bars) pain (NRS 0-10) during 3 months' participation in digitally delivered exercise and education treatment stratified by adherence. BL: baseline; NRS: numerical rating scale.

Weekly pain during the treatment stratified by baseline pain is illustrated in Figure 3. Those in a higher compared to lower tertile of baseline pain had a greater absolute and relative mean pain reduction at 3 months: NRS 2.8 (corresponding to a 38% relative change), 2.0 (37% relative change), and 0.9 (28% relative change) in the 3 tertiles, respectively (ANOVA *P*<.001 for the differences between all groups; Figure 3). Figure 4 illustrates weekly pain stratified by ≥90% versus <90% adherence to treatment.

### Associations With Reaching an MCIC in Pain at 3 Months

Bivariate analysis showed statistically significant associations between reaching an MCIC in pain and all sociodemographic characteristics with higher odds for the following: female compared to male (OR 1.4, 95% CI 1.3-1.5), age ≥65 compared to <65 years (OR 1.5, 95% CI 1.3-1.7), university educated compared to not (OR 1.5, 95% CI 1.3-1.6), and retired compared to working (OR 1.5, 95% CI 1.4-1.7). Variables indicating a worse baseline health were also statistically significantly associated with a higher odds of reaching an MCIC in pain as were the treatment-related variables of high motivation and high adherence (Table 3).

Multivariate analysis showed positive associations between reaching an MCIC in pain and high baseline pain (OR 1.9, 95% CI 1.6-2.1), high adherence (OR 1.5, 95% CI 1.3-1.8), and high motivation (OR 1.2, 95% CI 1.0-1.4). Further, we found negative associations for wish for surgery (OR 0.6, 95% CI 0.5-0.9) and pain in other joints (OR 0.9, 95% CI 0.7-0.9; Table 3).

**Table 3:** Variables associated with reaching a minimal clinically important change in LBP^a^ at 3-month follow-up (N=2593).

		Bivariate associations		Adjusted/multivariable associations	
		OR^b^ (95% CI)	*P* value	OR (95% CI)	*P* value
**Sociodemographic characteristics**					
	Sex (female)	1.4 (1.3-1.5)	<.001	0.9 (0.8-1.1)	.42
	Age (≥65 years)	1.5 (1.3-1.7)	<.001	0.9 (0.7-1.2)	.34
	Educational level (university)	1.5 (1.3-1.6)	<.001	1.1 (1.1-1.3)	.14
	Occupational status (retired)	1.5 (1.4-1.7)	<.001	1.2 (0.9-1.5)	.27
**Health-related characteristics**					
	BMI (>25)	1.5 (1.3-1.6)	<.001	1.1 (1.1-1.3)	.18
	Baseline LBP (>5 NRS)	2.0 (1.7-2.3)	<.001	1.9 (1.6-2.1)	<.001
	Having radiating pain (yes)	1.4 (1.3-1.6)	<.001	0.9 (0.8-1.1)	.37
	Pain medications (yes)	1.4 (1.3-1.6)	<.001	1.1 (0.8-1.2)	.99
	Wish for surgery	1.1 (0.8-1.6)	.5	0.6 (0.5-0.9)	.02
	Pain in other joints	1.3 (1.2-1.5)	<.001	0.9 (0.7-0.9)	.01
	Depression/anxiety	1.4 (1.2-1.5)	<.001	0.9 (0.8-1.1)	.24
	General health, NRS^c^ (0-10), (above mean)	1.5 (1.3-1.7)	<.001	1.1 (0.9-1.3	.36
	Physical activity ≥150 min/week	1.3 (1.2-1.5)	<.001	0.9 (0.8-1.0)	.16
**Treatment-related characteristics**					
	High motivation to start treatment (NRS=10)	1.6 (1.4-1.7)	<.001	1.2 (1.0-1.5)	.02
	≥90% adherence to treatment	1.8 (1.6-2.0)	<.001	1.5 (1.3-1.8)	<.001
	Number of interactions with PT,^d^ (above mean)	1.5 (1.3-1.7)	<.001	1.1 (0.9-1.3)	.29
	Participated in peer group (yes)	1.4 (1.2-1.6)	<.001	0.9 (0.8-1.1)	.33

^a^LBP: low back pain.

^b^OR: odds ratio.

^c^NRS: numerical rating scale.

^d^PT: physiotherapist.

### Associations With a Change From No to Yes for PASS (–To+)

Bivariate analysis showed statistically significantly associations between all sociodemographic characteristics and reporting PASS(–to+) but in opposite directions to associations seen in relation to reaching an MCIC in pain: female compared to male (OR 0.6, 95% CI 0.6-0.7), age ≥65 compared to <65 years (OR 0.6, 95% CI 0.6-0.7), university educated compared to not (OR 0.6, 95% CI 0.5-0.7), and retired compared to working (OR 0.6, 95% CI 0.6-0.7). Variables indicating a worse baseline health were associated with lower odds for reporting PASS(–to+). We found no association between reaching MCIC in pain and reporting PASS(–to+) in the bivariate analysis (Table 4).

Multivariable analysis showed a positive association between reporting PASS (–to+) and reaching an MCIC in pain (OR 4.1, 95% CI 3.4-5.1). Further, we found negative associations for wish for surgery (OR 0.3, 95% CI 0.2-0.5), high baseline pain (OR 0.5, 95% CI 0.4-0.6), depression or anxiety (OR 0.7, 95% CI 0.6-0.9), and high BMI (OR 0.8, 95% CI 0.7-1.0). We could not find that adherence was associated with PASS(–to+), but high motivation and high education were associated with a lower odds of PASS(–to+; Table 4).

**Table 4:** Variables associated with a change from no to yes for patient acceptable symptom state (PASS–to+) at 3-month follow-up (N=2080).

		Bivariate associations		Adjusted/multivariable associations	
		OR^a^ (95% CI)	*P* value	OR (95% CI)	*P* value
**Sociodemographic characteristics**					
	Sex (female)	0.6 (0.6-0.7)	<.001	1.0 (0.8-1.2)	.71
	Age (≥65 years)	0.6 (0.6-0.7)	<.001	0.9 (0.6-1.2)	.49
	Educational level (university)	0.6 (0.5-0.7)	<.001	0.7 (0.6-0.9)	.001
	Occupational status (retired)	0.6 (0.6-0.7)	<.001	1.0 (0.7-1.4)	.88
**Health-related characteristics**					
	BMI (>25)	0.6 (0.5-0.6)	<.001	0.8 (0.7-1.0)	.05
	Baseline LBP^b^ (>5 NRS^c^)	0.4 (0.4-0.5)	<.001	0.5 (0.4-0.6)	<.001
	Having radiating pain (yes)	0.6 (0.5-0.6)	<.001	0.9 (0.7-1.1)	.29
	Pain medications (yes)	0.5 (0.5-0.6)	<.001	0.9 (0.7-1.1)	.33
	Wish for surgery	0.2 (0.1-0.3)	<.001	0.3 (0.2-0.5)	<.001
	Pain in other joints	0.6 (0.5-0.6)	<.001	0.8 (0.7-1.0)	.06
	Depression/anxiety	0.5 (0.5-0.6)	<.001	0.7 (0.6-0.9)	.002
	General health, NRS (0-10; above mean)	0.8 (0.7-0.9)	<.004	1.2 (1.0-1.5)	.09
	Physical activity ≥150 min/week	0.6 (0.6-0.8)	<.001	1.0 (0.8-1.2)	.84
**Treatment-related characteristics**					
	High motivation to start treatment (NRS=10)	0.6 (0.6-0.7)	<.001	0.8 (0.6-1.0)	.02
	≥90% adherence to treatment	0.6 (0.5-0.6)	<.001	1.0 (0.8-1.3)	.74
	Number of interactions with PT,^d^ (above mean)	0.6 (0.5-0.6)	<.001	0.8 (0.7-1.1)	.05
	Participated in peer group (yes)	0.6 (0.6-0.7)	<.001	1.0 (0.8-1.2)	.90
	Reaching an MCIC^e^ in LBP at 3 months	1.0 (0.9-1.1)	<.84	4.1 (3.4-5.1)	<.001

^a^OR: odds ratio.

^b^LBP: low back pain.

^c^NRS: numerical rating scale.

^d^PT: physiotherapist.

^e^MCIC: minimal clinically important change.

## Discussion

### Principal Findings

Participants in this digitally delivered treatment for subacute or chronic LBP reduced their pain at 3-month follow-up, and 58.50% (1517/2593) reported an MCIC in pain. We found no difference in pain reduction in relation to sociodemographic characteristics, but those with high baseline pain, high motivation, and high adherence to treatment were more likely to reach an MCIC in pain, while those who at treatment start reported wish for surgery or had pain in other joints were less likely.

### Pain Reduction in Comparison to Prior Work

The pain reduction seen in this study is larger than that reported in initial digital self-management programs for LBP [13-15] but in line with recent apps with more complex ICT features and exercise support [17]. Baseline pain and disability among participants in our study (NRS pain around 5, ODI 26) was similar to those in previous digital and face-to-face interventions [17,33], but the mean age in this cohort was around 20 years higher compared to other digital interventions [17]. Our results suggest that digitally delivered treatment programs may show similar results in older adults with more complex health problems as in younger populations.

Consistent with those of higher age, a majority reported problems from other joints indicating that symptoms and age-related changes are worse in our sample compared to those reported in previous studies [17]. An encouraging finding is, however, that we did not find pain medications, high BMI, or depression or anxiety to be associated with a lower odds of reaching an MCIC in pain. This is in contrast with previous research suggesting lower BMI and low depression or anxiety scores at baseline to be associated with a more rapid decrease in pain [34] but is in line with a recent paper on middle-aged participants with multimorbidity and co-occurring MSK pain [35]. Few participants in our study reported that they wished to undergo surgery due to their LBP, but those who did were less likely to reach an MCIC in pain. Findings such as the ones reported here further reinforce the possibility that digital treatment programs can reduce pain at clinically important levels for older persons with more complex health problems, but people that report wish for surgery might need further attention.

### Adherence to Treatment

The high adherence in our study compared to that seen in other studies, and specifically in those being retired and of higher age, matched another report where older adults were less likely to drop out [34]. A previous study from our research group reported a mean adherence of 75% to recommended exercises for participants with hip or knee osteoarthritis staying in the treatment for 6 months [22], suggesting that high adherence rates can be maintained with support from a digitally delivered treatment program during longer periods. Frequency and duration of exercises, how adherence is measured, and what is considered a high adherence varies between studies [34], making comparisons between reports challenging. There is no conceptualization of adherence, but our chosen limit of 80% of activities performed during the treatment is in line with a systematic review of therapeutic exercise for MSK that reported 80%-99% of the recommended exercise dose as the most common limit for satisfactory adherence [29]. Given that we had valid data logged through the app, we were able to complete subanalyses on different adherence rates, finding a benefit of those with ≥90% adherence.

The dropout rate of 26% at 3 months was similar to that of other digitally delivered LBP treatment programs where dropouts have varied between 20% and 28% [17]. One digital program [36] reported substantial and increasing dropout rates during the treatment, with more than 80% dropping out before 12 weeks. Through app developments with systematically collected user feedback, dropouts could be reduced [37].

The association between adherence and pain reduction was not linear in this study in contrast to what has been shown in other studies [34,37]. One possible explanation might be that our program included a short duration and high frequency intervention (5-10 minutes/day) and not a longer duration and lower frequency (eg, 30 minutes 3 times/week) seen in most other programs. However, causality cannot be defined in an observational study such as the present one. It is well known that those with positive outcomes may adhere to treatment to a higher degree, while those not improving are more prone to missing out on exercises. However, a qualitative analysis in people participating in a digital program for hip and knee osteoarthritis revealed that reduced pain could also be a reason for lower adherence or not continuing with the program [8].

### MCIC and PASS

What constitutes an MCIC in pain probably varies from person to person, between conditions and treatments, and across different life and disease courses. Baseline pain severity has an impact, as a lower baseline pain score gives less room for change. The comparatively low proportion reaching MCIC in pain in our study, compared to those in recent studies that used a similar cutoff [34,38], may be related to participants in those studies being younger and at working age. Another way to estimate participant-relevant improvements is PASS. The proportion reporting PASS (+) at 3 months in our study is similar to that in a face-to-face randomized controlled trial when using an anchoring question of self-rated health and not the gold standard question used in our study [39].

MCIC reflects the concept of improvement (feeling better), while PASS deals with the concept of partial symptom remission or well-being (feeling good). We could not find that reaching an MCIC in pain was associated with reporting PASS(–to+) in bivariate analysis, but there was an association in the multivariable analysis. Those with high baseline pain and worse health were, in both bi- and multivariable analyses, less likely to report PASS, indicating that an MCIC of 2 points or 30% in pain is not enough to report “feeling good” for these people. Interestingly, we could not find an association between adherence and PASS(–to+). To our knowledge, there are no previous studies on the associations with PASS(–to+) after exercise treatment. Future studies on how factors such as duration of symptoms, expectations to treatment, and psychosocial aspects influence PASS would be of interest.

### Strengths and Limitations

The strengths of this study are that the treatment program is part of the health care system in Sweden and therefore includes people seeking care on their own. Another strength is the use of structured assessments of outcomes and adherence rates in a relatively large cohort.

There are limitations to consider. First, this was an observational study without a control group, and we cannot discern between specific and placebo treatment effects or natural fluctuations in symptoms. However, stratifying participants into different pain levels at treatment start showed that weekly improvement occurs similarly for all participants with no increasing pain in those with lower starting pain during the 3-month period. Second, for ethical reasons, we cannot say if those not giving consent to research differ in characteristics and outcomes in a way that could have influenced the results. Third, people seeking digitally delivered treatment may differ in several unknown ways, such as being more highly educated, compared to people participating in face-to-face treatments and compared to the total population, which may challenge external validity. Fourth, we only have follow-up data for a 3-month treatment period and can therefore not determine whether improvements can be sustained after the treatment.

### Conclusions

We found a clinically important reduction in pain for 58.50% (1517/2593) of participants after a 3-month digital treatment program for individuals with subacute or chronic LBP. We found no association with sociodemographic characteristics, but those with high baseline pain and high adherence were more likely to reach an MCIC in pain, while those wishing to undergo surgery or with pain in other joints at baseline were less likely to do so. Our findings suggest that digital treatment programs can reduce pain at clinically important levels for people with high adherence to treatment, but that those with such severe LBP problems that they wish to undergo surgery may benefit from additional support.

## Appendix

Multimedia Appendix 1 An example of what the app looks like when a patient enters the treatment.

Multimedia Appendix 2 Supplementary tables and figures.

## Abbreviations

ANOVA: analysis of variance
ICT: information and communication technology
JA: Joint Academy
LBP: low back pain
MCIC: minimal clinically important change
MSK: musculoskeletal
NRS: numeric rating scale
ODI: Oswestry Disability Index
OR: odds ratio
PASS: patient acceptable symptom state
PT: physiotherapist
STROBE: Strengthening the Reporting of Observational Studies in Epidemiology
