# Supplementary material for: Digitally Delivered Exercise and Education Treatment Program for Low Back Pain: Longitudinal Observational Cohort Study
Source: JMIR Rehabil Assist Technol. 2022 Jun 21;9(2):e38084. doi: 10.2196/38084 (PMC9257621; doi:10.2196/38084)
Supplement: Multimedia Appendix 1 [file rehab_v9i2e38084_app1.pdf]

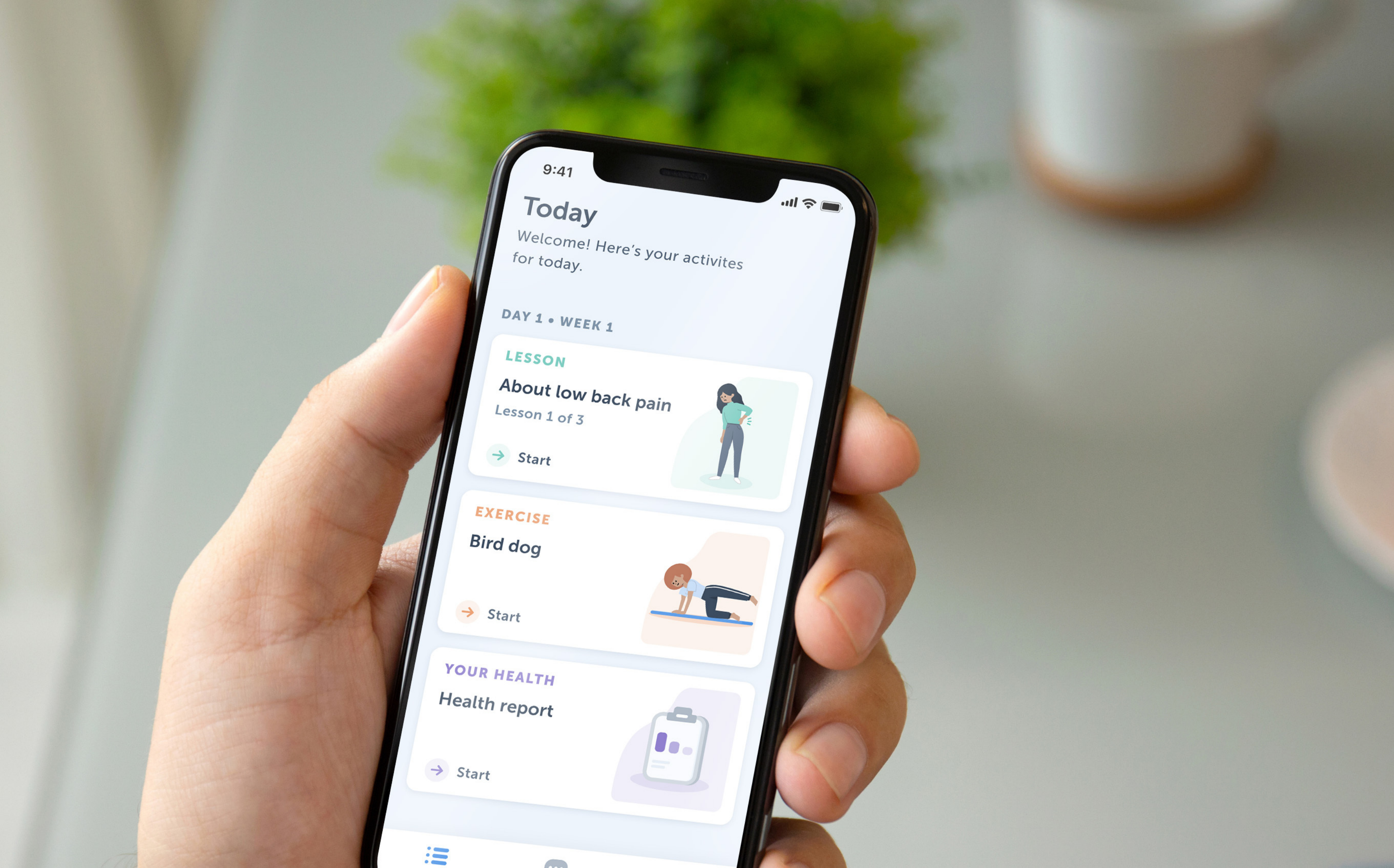
9:41
Today
Welcome! Here's your activites for today.
DAY 1 • WEEK 1
LESSON
About low back pain
Lesson 1 of 3
Start
EXERCISE
Bird dog
Start
YOUR HEALTH
Health report
Start
