# Supplementary material for: Digitally Delivered Exercise and Education Treatment Program for Low Back Pain: Longitudinal Observational Cohort Study
Source: JMIR Rehabil Assist Technol. 2022 Jun 21;9(2):e38084. doi: 10.2196/38084 (PMC9257621; doi:10.2196/38084)
Supplement: Multimedia Appendix 2 [file rehab_v9i2e38084_app2.doc]

**Multimedia appendix 2:** Supplementary Tables and figures

**Supplementary table 1**. Characteristics of participants in digitally delivered exercise and education treatment for low back pain, stratified by ≥90 % versus <90% adherence during the treatment, and for persons dropping out before 3-month follow-up

|  | ≥90% adherence (n=1300) | <90% adherence (n=1293) | *P*-value for difference | Drop outs before 3-month follow up (n=927) | *P*-value for difference  between drop-outs and total sample |
| --- | --- | --- | --- | --- | --- |
| **Sociodemographic characteristics** | | | | | |
| Female, n (%) | 962 (74.00) | 953 (73.70) | .864 | 645 (69.58) | .012 |
| Age (years), mean  (sd) | 65.2 (10.0) | 60.8 (11.6) | <.001 | 59.2 (12.6) | <.001 |
| Educational level, n (%)  -Haven't graduated high school  -High school  -College / University degree | 129 (9.92)  466 (35.85)  705 (54.23) | 92 (7.12)  487 (37.66)  714 (55.22) | .063  .422  .418 | 94 (10.14)  378 (40.78)  455 (49.08) | .733  .524  <.001 |
| Occupational status, n (%)  -Working  -Retired  -Other | 446 (34.31)  789 (60.69)  65 (5.00) | 647 (50.04)  522 (40.37)  124 (9.59) | <.001  <.001  <.001 | 502 (54.15)  339(36.57)  86 (9.28) | <.001  <.001  .053 |
| **Baseline health-related characteristics** | | | | | |
| Body mass index (kg/m2 ), mean (sd) | 25.9 (4.1) | 27.0 (4.7) | <.001 | 27.1 (4.8) | <.001 |
| Baseline pain, NRS (0-10), mean (sd) | 4.9 (1.9) | 4.8 (1.8) | .211 | 5.1 (2.0) | <.001 |
| Reported radiating pain (>0 NRS), n (%) | 827 (63.60) | 803 (62.10) | .356 | 578 (62.35) | .700 |
| Pain medications for back pain during last month, n (%) | 615/1023  (60.12) | 637 (58.66) | .165 | 567 (63.07) | <.001 |
| So severe problems that wish to undergo surgery, yes, n (%) | 64 (4.92) | 74 (5.72) | .364 | 85 (9.17) | <.001 |
| Pain in other joints, n (%) | 988 (76.00) | 968 (74.86) | .502 | 640 (69.04) | <.001 |
| Depression/anxiety (any problem =level 2-5 EQ-5D-5L), n (%) | 620 (47.69) | 731 (56.54) | <.001 | 553 (59.66) | <.001 |
| General health, NRS (0-10), mean (sd) | 6.4 (1.6) | 6.0 (1.7) | <.001 | 5.9 (1.8) | <.001 |
| Physical activity level, ≥150 min/week, n (%) | 568 (43.69) | 497 (38.44) | .007 | 296 (31.93) | <.001 |
| Motivation/readiness ruler to start exercising, (NRS 0-10, not at all to extremely), mean (sd) | 9.4 (1.1) | 9.1 (1.4) | <.001 | 8.8 (1.7) | <.001 |
| **Treatment-related characteristics** | | | | | |
| Number of chat interactions with the physiotherapist during the treatment | | | | | |
| Messages received from the PT, mean (sd) | 22 (13) | 21 (10) | .003 | 11 (7) | <.001 |
| Messages sent to the PT, mean (sd) | 9 (8) | 9 (7) | .733 | 5 (4) | <.001 |
| Participated in peer support group, n (%) | 464 (35.69) | 406 (31.40) | .014 | 91 (9.82) | <.001 |
| Adverse events (yes/no), n (%) | 26 (2.00) | 37 (2.86) | .154 | Not applicable | Not applicable |
| Out of which: | | | | | |
| Pain more than 24 h, n (%) | 5 (19.23) | 11 (30.56) |  | Not applicable | Not applicable |
| Fall/Injury, n (%) | Not applicable | 1 (2.78) |  | Not applicable | Not applicable |
| Other, n (%) | 21 (80.77) | 24 (66.67) |  | Not applicable | Not applicable |

**Supplementary figure 1.** Attrition rates/dropouts during 3 months treatment for all persons with baseline data (n=3520 at baseline)

**
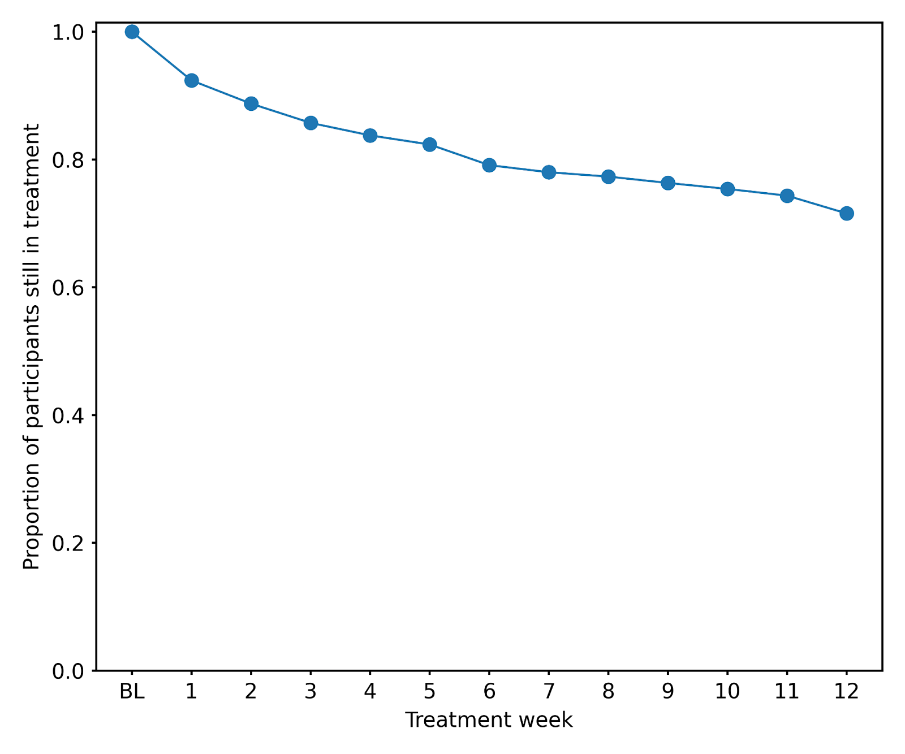
**
